# Associations between maternal depressive symptoms and selective serotonin reuptake inhibitor antidepressant treatment on internalising and anxiety behaviours in children: 12-year longitudinal study

**DOI:** 10.1192/bjo.2022.623

**Published:** 2023-02-01

**Authors:** Sarah M. Hutchison, Ursula Brain, Ruth E. Grunau, Boris Kuzeljevic, Mike Irvine, Louise C. Mâsse, Tim F. Oberlander

**Affiliations:** Department of Pediatrics, University of British Columbia, Vancouver, Canada; (deceased), Department of Pediatrics, University of British Columbia, Vancouver, Canada; School of Population and Public Health, University of British Columbia, Vancouver, Canada; Department of Pediatrics, University of British Columbia, Vancouver, Canada; and School of Population and Public Health, University of British Columbia, Vancouver, Canada;

**Keywords:** Perinatal period, paediatrics, anxiety, internalising, maternal mood

## Abstract

**Background:**

Prenatal selective serotonin reuptake inhibitor (SSRI) antidepressant exposure is associated with increased internalising and anxious behaviours in young children; whether this continues into early adolescence is unknown. Also, it is not well established whether it is the *in utero* exposure to SSRIs or the underlying maternal mood that contributes more to these associations.

**Aims:**

To examine associations between maternal depressive symptoms, prenatal SSRI antidepressant treatment and internalising and anxiety behaviours from childhood into pre-adolescence.

**Method:**

From a prospective longitudinal cohort, measures of maternal depressive symptoms and SSRI use and child outcomes (*n* = 191 births) were obtained from the second trimester to 12 years. Maternal reports of internalising and anxiety behaviours in children were obtained at 3, 6 and 12 years.

**Results:**

Multilevel mixed-effects models revealed that maternal depressed mood at the third trimester assessment, not prenatal SSRI exposure, was associated with longitudinal patterns of higher levels of internalising and anxiety behaviours across childhood from 3 to 12 years of age. At each age, hierarchical regressions showed that maternal mood at the third trimester, compared with current maternal depression or prenatal SSRI exposure, explained a greater proportion of the variance in internalising and anxiety behaviours.

**Conclusions:**

Even with prenatal SSRI treatment, maternal depressed mood during the third trimester still had an enduring effect as it was associated with increased levels of internalising and anxiety behaviours across childhood and into early adolescence. Importantly, we found no evidence of a ‘main effect’ association between prenatal SSRI exposure and internalising and anxiety behaviours in children.

Approximately 10–20% of women experience mood disturbances (depression and/or anxiety) during the perinatal period and up to one-third of these women are treated with an SSRI.^1^ SSRIs are prescribed during and following pregnancy to treat or even prevent perinatal depression, with the expectation of benefit to mothers and their children. However, prenatal SSRI exposure has been associated with increased internalising and anxious behaviours in children of mothers treated with an SSRI during pregnancy,^2–4^ raising critical questions about what underlies apparent associations between prenatal SSRI treatment and child behaviour, especially in outcomes beyond infancy and early childhood.^5^

## Prenatal SSRI exposure and behavioural outcomes

SSRIs readily cross the placenta and fetal blood–brain barrier^6,7^ and given that SSRIs potentiate presynaptic levels of the key neurodevelopmental neurotransmitter serotonin (5-HT) involved with early brain growth and development,^8^ such exposure raises concerns about whether altered serotonin levels during critical periods of early brain development are associated with long-term behavioural outcomes. Prolonged increased levels of 5-HT in the fetal brain, via negative feedback, constrain development of the 5-HT circuitry, reduce serotonergic tone and ultimately lower 5-HT levels during development.^9,10^ In animal models, increased central 5-HT levels (e.g. by genetic variations, 5-HT agonists or SSRI exposure) paradoxically constrain the development of the 5-HT system, leading to increased anxiety-like behaviours later in life.^11^

In humans, higher levels of internalising (anxious and depressive) behaviour in 3- to 5-year-olds have been reported in children with prenatal SSRI exposure;^12^ however, that study did not control for pre- or post-natal maternal mood disturbances. A recent Danish population study reported that maternal prenatal antidepressant treatment was associated with an increased risk of affective disorders across an 18-year follow-up period; however, as similar associations were also found in children whose fathers continued antidepressant treatment across the pregnancy period, these may be related to parental psychopathology, rather than in utero exposure to antidepressants.^13^ In other studies, even when controlling for pre- and postnatal depressive symptoms, internalising behaviours in 3-year-olds^14^ and 6-year-olds^3^ remained associated with prenatal SSRI exposure. Conversely, other studies failed to find significant differences in internalising behaviour between SSRI- (or antidepressant) exposed and non-exposed groups.^15–18^ Group differences were also not found in relation to conduct problems and affective problems in children less than 6 years old.^19,20^ Importantly, these behavioural outcomes were related to higher levels of either prenatal mental disturbances,^15^ postnatal anxiety and depression symptoms at the time of testing^16,20^ or a combination of pre- and postnatal depressive symptoms.^17–19^

A few studies that have also examined anxiety-specific outcomes in children. Prenatal antidepressant exposure has been associated with higher anxiety in 3-year-olds compared with non-exposed siblings, controlling for pre- and postnatal maternal anxiety and depressive symptoms.^2^ Similar results were also found in a longitudinal study that followed mothers and their children from fetal life *in utero* to 6 years of age, which showed that children with prenatal SSRI exposure had increasing levels of anxiety behaviours compared with children without SSRI exposure, even when controlling for maternal depressive symptoms.^3^ Another longitudinal study found that only SSRI exposure during late pregnancy (28 weeks or more) was associated with increased levels of anxiety at 5 years of age, but not at 18 months or 3 years of age, even when prenatal maternal anxiety and depressive symptoms were taken into account.^21^ Another study reported no evidence of associations between SSRI exposure and diagnosis of an anxiety disorder in young children;^22^ however, again, pre- and postnatal measures of maternal anxiety or depressive symptoms were not included in the study.

These mixed results may be related to inconsistent measures of prenatal^16,20^ and current maternal mood disturbances,^15^ which commonly constrains our ability to distinguish the effect of maternal mood disturbances from SSRI exposure in studying internalising and anxiety behaviours in children. Distinguishing the effect of the SSRI from that of maternal mood during pregnancy, which has also been associated with altered child behaviour, namely the issue of ‘confounding by indication’, remains a key challenge.^5^ Specifically, maternal psychopathology may assert its effects through other factors, such as environmental stress and less than optimal parenting practices,^5^ thereby contributing to the development of internalising and anxiety behaviours. In addition, prenatal SSRI exposure has been associated with increased internalising and anxious behaviours in young children; however, whether this continues into early adolescence is unknown. The current study was undertaken to address these gaps in the literature.

## The hypothesis

This study sought to examine the longitudinal patterns of internalising and anxious behaviours from 3 to 12 years of age and determine the relative contributions of maternal prenatal depressive symptoms and current depressive symptoms and of prenatal SSRI exposure to child internalising and anxious behaviours at 3, 6 and 12 years. Internalising behaviours and anxious behaviours were both included as a reflection of a broader range of the child's emotional state (i.e. internalising behaviours) that might include depressive, anxiety and somatic symptoms, apart from anxiety alone. We expected that across childhood and into early adolescence, prenatal SSRI exposure would be associated with persistently higher levels of internalising and anxiety behaviours, even accounting for prenatal and current maternal depressive symptoms. We reported outcomes separately for internalising and anxiety to allow direct comparisons with previous research.

## Method

### Participants

Mothers were recruited during their second trimester as part of a larger longitudinal cohort study from community (family practice, midwifery) and tertiary referral clinics (reproductive mental health) examining the developmental effects of prenatal exposure to SSRI antidepressants. Participants comprised a volunteer sample of pregnant women who were depressed and treated with an SSRI, depressed and not treated with an SSRI and not depressed or treated with an SSRI. Participants were either diagnosed with a mood disorder and SSRI-treated based on clinical need prior to or during pregnancy, were depressed and chose not to treat their depression with antidepressants or were not depressed during pregnancy. All SSRI-treated mothers had a diagnosed mood disorder of depression and/or anxiety (Table 1) and had started taking medications based on clinical need. Pharmacotherapy included a range of serotonin reuptake inhibitors and serotonin–noradrenaline reuptake inhibitors, collectively referred to in this study as SSRIs. For the present study we relied on self-report of drug use as shown in Table 1 (prenatal treatment days); however, drug levels in this cohort have been reported in a previous publication.^23^ Among mothers using SSRIs, the most common drug treatment was paroxetine (Table 2) and the mean duration of exposure for all SSRIs spanned most or almost the entire pregnancy.

**Table 1:** Descriptive statistics for key variables in mothers^a^

	Not treated with an SSRI (total *n* = 115)	*n*	SSRI treated (total *n* = 76)	*n*	*T* or χ^2^	*P*	Effect size (*r*)^c^ or phi
**Maternal characteristics**							
Gestation at second trimester, weeks: mean (s.d.)	26.65 (3.10)	108	24.98 (4.48)	73	2.98	0.003	0.22
Diagnosis at study entry (depression), *n* (%)	23 (20.2%)	114	62 (81.6%)	76	69.54^b^	< 0.001	0.61^d^
Diagnosis at study entry (anxiety), *n* (%)	28 (24.6%)	114	54 (71.0%)	76	40.18	< 0.001	0.46^d^
Age at delivery, mean (s.d.)	33.65 (5.04)	110	32.70 (5.43)	76	1.22	0.223	0.09
Education, years: mean (s.d.)	17.73 (3.34)	113	16.36 (3.21)	76	2.83	0.005	0.20
Ethnicity, *n* (%)					1.38	0.495	0.09^d^
White	90 (47.1%)		60 (31.4%)				
Asian	12 (6.3%)		5 (2.6%)				
Other	12 (6.3%)		11 (5.8%)				
Prenatal SSRI treatment, days: mean (s.d.)	0 (0.00)	115	235 (67.87)	75	−37.21	< 0.001	0.94
Prenatal smoking, mean (s.d.)		108		74		0.673^e^	
No	105 (97.2%)		72 (97.3)				
Yes	3 (2.8%)		2 (2.7)				
Prenatal alcohol use	3.42	109	4.05	75	−0.55	0.585	0.04
HAMD second trimester, mean (s.d.)	6.68 (6.58)	109	11.90 (6.75)	73	−5.20	< 0.001	0.36
HAMD third trimester, mean (s.d.)	5.58 (5.58)	102	10.12 (5.87)	73	−5.26	< 0.001	0.37
HAMD at 6 years, mean (s.d.)	6.28 (5.67)	72	10.98 (6.91)	51	−4.14	< 0.001	0.35
HAMD at 12 years, mean (s.d.)	5.63 (5.70)	57	9.84 (6.41)	31	−3.17	< 0.001	0.32
BDI at 3 years, mean (s.d.)	4.22 (4.39)	88	8.12 (5.88)	59	−4.59	< 0.001	0.36
BDI at 12 years, mean (s.d.)	6.79 (6.33)	14	7.50 (5.54)	6	−0.24	0.814	0.06

SSRI, selective serotonin reuptake inhibitor; HAMD, Hamilton Rating Scale for Depression; BDI, Beck Depression Inventory.

a. Clarifications: Asian, those who self-identified as Oriental; White, those who self-identified as Caucasian; Other, those who self-identified as the following: Indian (*n* = 6), Latin (*n* = 5), Oriental/White (*n* = 3), Native North American Indian (*n* = 2), and one mother in each of the following self-identifying categories: Filipino/Polynesian, Black, Greek, Israeli, Eurasian, Jewish, West Indian, and preferred not to respond; SSRI treatment days, number of days of prenatal SSRI treatment (range: 41–294 days), no information was collected on timing of treatment but most mothers were taking SSRIs at the time of conception; prenatal smoking: Yes means <20 cigarettes per day; prenatal alcohol use, maternal alcohol consumption in single drinks during entire pregnancy; CBCL above threshold, number and percentage of participants with a *T*-score of 70 or higher on the CBCL; SCARED above threshold risk, number and percentage of participants with a total score of 25 or higher, which may indicate the presence of an anxiety disorder.

b. χ^2^ value.

c. Cohen (1988) proposed guidelines of effect sizes for small, medium and large effects for both individual differences (Pearson's *r* = 0.10, 0.30 and 0.50, respectively) and group differences (Cohen's *d* or Hedges' *g* = 0.20, 0.50 and 0.80).

d. Phi value.

e. As the number of the computed expected clinical cases in the SSRI group was less than 5, Fisher's exact test is reported; no effect size can be computed.

**Table 2:** Specific maternal selective serotonin reuptake inhibitors (SSRIs), prenatal dose and length of prenatal exposure

SSRI type	Mothers taking SSRI, *n* (%)	Days of prenatal exposure, mean (s.d.)	Range (days of exposure)
Paroxetine	22 (28.9)	229.41 (68.98)	61–292
Fluoxetine	11 (14.5)	212.27 (74.32)	106–294
Sertraline	12 (15.8)	220.45 (84.45)	65–284
Venlafaxine	16 (21.1)	257.50 (58.66)	41–287
Citalopram	14 (18.4)	246.07 (57.32)	94–290
Escitalopram	1 (0.5)	274.00 (.)	274
All SSRIs	76 (100)	235.28 (67.87)	41–294

Inclusion criteria for this study were singleton pregnancy, confirmed gestational age, ability to give informed consent and no fetal anomalies detected by ultrasound. Exclusion criteria were bipolar disorder, illicit drug use and significant maternal medical, obstetrical or fetal conditions. Written informed consent was obtained from all mothers. The authors assert that all procedures contributing to this work comply with the ethical standards of the relevant national and institutional committees on human experimentation and with the Helsinki Declaration of 1975, as revised in 2008. All procedures involving human participants were approved by the University of British Columbia/Children's & Women's Health Centre of British Columbia Research Ethics Board (UBC C&W REB).

We had 254 mothers contact us to express interest in the study. Of these, 21 were excluded from the study (too far along in pregnancy (*n* = 14), miscarried (*n* = 4), twin pregnancy (*n* = 2), recreational drug user (*n* = 1)) and 42 chose to withdraw from the study (no reason given (*n* = 22), lived or delivered outside of study area (*n* = 9), too much time commitment (*n* = 7), ultrasound perceived as dangerous (*n* = 4)). Of the original 191 mothers studied up to delivery, outcomes for this study included 147 mother–child dyads at 3 years (61 male children, 86 female), 145 dyads at 6 years (65 males, 80 females) and 112 dyads at 12 years (47 males, 65 females). Of the original 191 mothers (76 mothers with prenatal SSRI treatment), there were 147 mothers at 3 years (59 mothers with prenatal SSRI treatment), 145 mothers at 6 years (56 mothers with prenatal SSRI treatment) and 112 mothers at 12 years (38 mothers with prenatal SSRI treatment). In terms of which mothers continued SSRI treatment over time, of the original 76 prenatal SSRI-treated mothers, at 3 years 43 of 59 (72.9%), at 6 years 39 of 56 (69.6%) and at 12 years 29 of 38 (76.3%) continued SSRI treatment. Of the original 115 mothers not depressed/not treated with an SSRI during pregnancy, 8 of 88 at 3 years (9.1%), 7 of 89 (7.9%), at 6 years and 11 of 74 (14.9%) at 12 years subsequently started an SSRI. Differences in prenatal depression symptoms between mothers who participated and mothers who dropped out or where we were not able to obtain child behaviour reports were examined. Mothers who participated at 12 years were less depressed prenatally (Hamilton Rating Scale for Depression score in the third trimester: mean 6.32, s.d. = 5.57) than mothers who withdrew or had missing data (mean 8.95, s.d. = 5.93; *t* = −3.14, *P* = 0.002). However, there was no significant difference in the proportion of mothers who had been treated with an SSRI during pregnancy and participated at 12 years compared with mothers who had been treated with an SSRI during pregnancy and who withdrew or had missing data (*χ*^2^(1) = 4.10, *P* > 0.05).

To examine possible bias in our study due to missing data, we conducted scenario-based quantitative bias analysis.^24^ We assumed the following scenarios for the two groups of children at 12 years of age (with and without SSRI exposure) who had dropped out or had missing data: (a) no children met the clinical threshold for anxiety symptoms, (b) 31.4% of children met the clinical threshold for anxiety symptoms (31.4% was selected as this is the estimated lifetime prevalence of any anxiety disorder among 13- to 14-year-olds based on data from 2001 to 2004 in the USA^25^) and (c) 100% of children met the clinical threshold for anxiety symptoms. These scenarios were selected to represent both extreme (0%, 100%) and reasonable (31.4% based on current research estimates) possibilities in our missing data. We also conducted the same analyses for internalising symptoms, but we selected 25% as the second scenario as there was no previous research estimating internalising symptoms in adolescents to draw from. Results showed that the number of children meeting the clinical threshold for anxiety and internalising would increase proportionally to the hypothetical scenario examined (details are given in the supplementary material, available at https://doi.org/10.1192/bjo.2022.623), suggesting that despite the attrition in our sample over time, our results would be similar to a complete cohort with no attrition.

### Measures

#### Maternal mood

Maternal depressed mood was assessed using the a clinician-rated measure, the Hamilton Rating Scale for Depression (HAMD)^26^ at the third trimester of pregnancy and at 6 and 12 years postpartum. The HAMD is a clinician-rated measure of depressive symptoms, with higher scores indicating higher levels of depression; total scores were used in the analyses. At 3 years, measures of depressive symptoms were obtained using the 21-item self-reported Beck Depression Inventory (BDI)^27^ as we did not have the resources to conduct in-person assessments at this time point. This multiple-choice self-report questionnaire is designed to assess the severity of depression, with higher scores indicating higher levels of depressive symptoms. The BDI is well established as being highly correlated with the HAMD at multiple time points.^28^ Maternal mood was treated as a continuous measure, reflecting a dimensional range of depressive symptoms.

#### Child internalising and anxiety behaviour

At 3 years and 12 years, maternal reports of internalising and anxiety problems in their children were obtained using the Child Behavior Checklist (CBCL) (for 1.5- to 5-year-olds^29^ and 6- to 18-year-olds^30^), a norm-referenced caregiver-completed rating scale that describes a child's functioning during the previous 6 months. All items are scored on a three-point Likert scale (0, not true; 1, somewhat or sometimes true; 2, very true or often true). All CBCL scales have a *T*-score mean of 50 and s.d. of 10 and different norms are provided for each gender across the age ranges of 6–11 years and 12–18 years. The CBCL yields a total problem score, externalising and internalising scores, and norm-referenced DSM-oriented scales, which include an anxiety problems scale. These DSM-oriented scales were created based on expert consensus of selected items from the CBCL and were developed to assist practitioners in the differential diagnostic process. The anxiety problems scale assesses symptoms of separation anxiety disorder, specific phobia and generalised anxiety disorder. There is substantial psychometric support for the various CBCL scales.^30,31^ For the current study, we used the internalising and anxiety problems *T*-scores, which are computed based on the gender and age of the child.

When their children were 6 years of age, mothers completed the MacArthur Health and Behavior Questionnaire (HBQ), which yielded measures of internalising, externalising, over-anxious and inattention behaviours. These measures were derived from the Ontario Child Health Study Measure, which maps onto items from the CBCL and DSM-III-R symptom criteria for internalising behaviours^32^ in children ages 4 to 8 years.^33^ We also used the Screen for Child Anxiety Related Emotional Disorders (SCARED),^34^ a child and parent self-report instrument, to compare the mother's report of anxiety with that of their child, to examine whether mothers treated with a prenatal SSRI over-reported internalising and anxiety behaviours in their children.

#### Statistical analyses

Group differences in maternal and child characteristics and outcomes were determined using *t*-tests or χ^2^-tests as appropriate. To determine longitudinal associations between predictor variables, multilevel mixed model analyses were used to examine repeated measures (at 3, 6 and 12 years) and group differences in child behaviours as this method prevented listwise deletion owing to missing data^35^ and enabled use of 160 mothers and their children from the original birth cohort (*n* = 191) where we had data from at least two time points. For these analyses, we used one continuous predictor variable (maternal mood at the third trimester), one categorical predictor variable (prenatal SSRI treatment: yes/no) and one interaction term (maternal mood at the third trimester × prenatal SSRI exposure). We chose maternal mood at the third trimester because this was the closest measurement during pregnancy relative to the other time points (at 3, 6 and 12 years). Standardised internalising and anxiety behaviour scores (*z*-scores) were derived from CBCL and HBQ internalising and anxiety behaviour measures at 3 and 12 years and at 6 years respectively. Two multilevel mixed models were used to assess whether the selected variables predicted internalising behaviour and to predict anxiety behaviour during the entire follow-up period.

To determine the relative contributions of each key exposure (prenatal depressed maternal mood, current maternal mood and SSRI exposure) on internalising behaviours at each age, three hierarchical regressions were conducted with internalising behaviour scores at 3, 6 and 12 years as the dependent variable. To determine the unique variance explained by each predictor (maternal mood at the third trimester, current maternal mood, SSRI exposure status) at each assessment (which is not possible using multilevel mixed model analyses), three hierarchical regressions were used separately, with internalising and anxiety measures at 3, 6, and 12 years as the dependent variables. Child age at the time of the study was entered at step 1, third trimester maternal HAMD scores were entered at step 2, current mood was entered at step 3 (e.g. for internalising measures at 3 years, HAMD scores at 3 years were entered; for internalising measures at 6 years, HAMD scores at 6 years were entered, etc.) and SSRI exposure status was entered at step 4.

There were missing maternal HAMD scores (e.g. third trimester: *n* = 4; 6 years: *n* = 22: 12 years: *n* = 23) and BDI scores (3 years: *n* = 8; 12 years: *n* = 90) as described above. Three mothers at the 12-year time point did not complete either the HAMD or the BDI. Missing values were imputed using an expectation maximisation algorithm to estimate missing data from key population parameter values (SYSTAT version 13 for Windows).

Order of entry of variables for the hierarchical regressions was based on previous cross-sectional research demonstrating that maternal mood was more associated with children's affective behaviour than was SSRI exposure.^15,17,18^ All assumptions necessary for multiple regression analyses to be conducted and considered valid were met (e.g. absence of multicollinearity for the two maternal mood measures, as the variance inflation factor (VIF) was <10 and tolerance was >0.2) and no outliers or influential cases were detected. Maternal mood at the third trimester was included as a key covariate in the multilevel mixed models and hierarchical regressions. Current maternal mood was treated as a predictor for the hierarchical regressions only at each time point (3, 6 and 12 years). All analyses were conducted using SPSS version 19 for Windows.

## Results

### Maternal characteristics

Mothers with prenatal SSRI treatment had persistently higher levels of depressive symptoms during the second and third trimesters, and at 3 years, 6 years and 12 years, compared with mothers without prenatal SSRI treatment (Table 1). Characteristics of mothers with and without prenatal SSRI treatment did not significantly differ regarding ethnicity, prenatal smoking and prenatal alcohol use.

Mothers in both groups had a range of depressive symptoms at each assessment. Mothers with prenatal SSRI treatment reported significantly higher depressive symptoms than mothers without SSRI treatment. All group HAMD means (<11) were below the typical clinical cut-off for a diagnosis of moderate depression (17–23) and severe depression (≥24)^36^; however, we were interested in a range of depressive symptoms and we did not limit our study only to mothers who met diagnostic criteria for depression at each assessment.

To examine the possibility that mothers with prenatal SSRI treatment were over-reporting internalising and anxiety behaviours in their children, intraclass correlation coefficients (ICCs) were used to evaluate the agreement between maternal reports of anxiety and their children's self-reports using the SCARED.^34^ The ICC provides an index of absolute agreement as it takes into account the ratio between participant variability and total variability.^37^ ICCs were computed for the whole sample and by subsample (SSRI exposure versus no SSRI exposure) to investigate whether there were differences in mother–child agreement depending on SSRI exposure. ICCs are classified as follows: <0.40 indicates poor to fair agreement; 0.41–0.60 moderate agreement; 0.61–0.80 good agreement; and 0.81–1.00 excellent agreement.^38^ At 12 years for the whole sample, the ICC between maternal report and their child's self-report for the SCARED total score was 0.62, *P* < 0.001 (*n* = 110). At 12 years among mothers who had been treated with an SSRI in pregnancy and their child's self-report for the SCARED total score, ICC = 0.76, *P* < 0.001 (*n* = 37). At 12 years among mothers without SSRI treatment and their child's self-report for the SCARED total score, ICC = 0.76, *P* < 0.001 (*n* = 73). In sum, these results suggest convergence of maternal and child self-reports of anxiety symptoms.

### Child characteristics

At the 3- and 6-year assessment points, SSRI-exposed children were significantly older than children without SSRI exposure (Table 3). There were no significant differences between children with and without SSRI exposure in relation to the number of females in each group. There were also no gender differences in children's internalising and anxiety behaviours at 3, 6 and 12 years (data not shown, all *P* in the range 0.243–0.984).

**Table 3:** Descriptive statistics for key variables in children

	No SSRI exposure (total *n* = 115)	*n*	SSRI exposure (total *n* = 76)	*n*	*T* or χ^2^	*P*	Effect size (*r*)^c^ or phi
**Child characteristics**							
Gestational age at birth, weeks: mean	39.75 (1.75)	110	39.05 (1.60)	75	2.80	0.006	0.20
Sex at birth (females), *n* (%)	58 (52.7%)	110	42 (55.2%)	75	0.19^a^	0.764	0.03^d^
Delivery mode (Caesarean section), *n* (%)	30 (23.7%)	110	22 (29.3)	75	0.09	0.760	0.02
Birth weight, g: mean (s.d.)	3492.05 (545.32)	110	3323.12 (517.61)	75	2.11	0.036	0.15
Birth length, cm: meand (s.d.)	51.65 (2.82)	109	50.61 (2.36)	75	2.63	0.009	0.19
Apgar score (1 min), mean (s.d.)	8.24 (1.39)	110	7.48 (1.63)	75	3.38	0.001	0.24
Apgar score (5 min), mean (s.d.)	8.99 (0.57)	110	8.80 (0.74)	73	1.99	0.048	0.15
Age at 3 years, mean (s.d.)	3.53 (0.57)	88	3.85 (0.63)	59	−3.22	0.002	0.26
Age at 6 years, mean (s.d.)	5.81 (0.59)	89	6.12 (0.79)	56	−2.73	0.007	0.22
Age at 12 years, mean (s.d.)	11.68 (1.11)	78	11.88 (1.17)	40	−0.65	0.518	0.06
CBCL score at 3 years, mean (s.d.)							
Internalising problems	45.88 (8.43)	88	49.56 (10.53)	59	−2.15	0.034	0.18
Anxiety problems, mean	51.73 (3.40)	88	52.20 (5.58)	59	−0.56	0.648	0.04
HBQ score at 6 years, mean (s.d.)							
Internalising symptoms	0.28 (0.21)	88	0.38 (0.26)	54	−2.58	0.011	0.21
Overanxious	0.36 (0.26)	88	0.48 (0.32)	54	−2.42	0.017	0.20
CBCL score at 12 years							
Internalising problems, mean (s.d.)	51.16 (10.40)	73	58.14 (10.91)	37	−3.35	0.001	0.31
Above threshold, *n* (%)	4 (5.5%)	73	6 (16.2%)	37		0.083^b^	
Anxiety problems, mean (s.d.)	54.89 (7.26)	73	58.70 (10.19)	37	−2.46	0.016	0.23
Above threshold, *n* (%)	6 (8.2%)	73	8 (21.6%)	37	3.97	0.068	0.19^d^
SCARED at 12 years							
Total score, mean (s.d.)	11.62 (9.48)	73	19.55 (13.42)	38	−3.62	< 0.001	0.33
Above threshold risk, *n* (%)	8 (11.0%)	73	11 (28.9%)	38	5.70^a^	0.031	0.23^d^
SCARED at 12 years: child self-report							
Total score, mean (s.d.)	16.59 (9.83)	73	19.84 (10.02)	37	−1.63	0.107	0.15
Above threshold risk, *n* (%)	15 (20.5%)	73	11 (29.7%)	37	1.15^a^	0.344	0.10^d^

SSRI, selective serotonin reuptake inhibitor; CBCL, Child Behavior Checklist; HBQ, Health Behavior Questionnaire; SCARED, Screen for Child Anxiety Related Emotional Disorders.

a. Clarifications: Asian, those who self-identified as Oriental; White, those who self-identified as Caucasian; Other, those who self-identified as the following: Indian (*n* = 6), Latin (*n* = 5), Oriental/White (*n* = 3), Native North American Indian (*n* = 2), and one mother in each of the following self-identifying categories: Filipino/Polynesian, Black, Greek, Israeli, Eurasian, Jewish, West Indian, and preferred not to respond; SSRI treatment days, number of days of prenatal SSRI treatment (range: 41–294 days), no information was collected on timing of treatment but most mothers were taking SSRIs at the time of conception; prenatal smoking: Yes means <20 cigarettes per day; prenatal alcohol use, maternal alcohol consumption in single drinks during entire pregnancy; CBCL above threshold, number and percentage of participants with a *T*-score of 70 or higher on the CBCL; SCARED above threshold risk, number and percentage of participants with a total score of 25 or higher, which may indicate the presence of an anxiety disorder.

b. χ^2^ value.

c. Cohen (1988)^39^ proposed guidelines of effect sizes for small, medium and large effects for both individual differences (Pearson's *r* = 0.10, 0.30 and 0.50, respectively) and group differences (Cohen's *d*or Hedges' *g* = 0.20, 0.50 and 0.80).

d. Phi value.

### Children's internalising behaviours across childhood

Multilevel mixed model analyses (*n* = 160) investigated the impact of maternal mood at the third trimester, SSRI exposure and an interaction between maternal mood at the third trimester and SSRI exposure. Results showed that maternal mood at the third trimester was associated with increased internalising behaviours over time (Table 4). Neither SSRI exposure alone nor an interaction between SSRI exposure and third trimester maternal mood contributed significantly to the model.

**Table 4:** Estimated fixed effects of predictors for internalising and anxiety behaviours in children with and without prenatal exposure to selective serotonin reuptake inhibitors (SSRIs)^a^

Parameter	Estimate	s.e.	d.f.	*t*	*P*	95% CI	
						Lower	Upper
Internalising							
Intercept	−0.34	0.18	139.26	−1.82	0.071	−0.72	0.03
Maternal mood at the third trimester	0.07	0.02	137.27	4.04	< 0.001	0.03	0.10
SSRI exposure	−0.01	0.22	137.98	−0.06	0.955	−0.43	0.41
Interaction (maternal mood at the third trimester × SSRI exposure)	−0.03	0.02	146.23	−1.35	0.180	−0.074	0.01
Anxiety							
Intercept	−0.28	0.18	140.91	−1.54	0.125	−0.64	0.07
Maternal mood at the third trimester	0.05	0.02	138.50	3.36	0.001	0.02	0.08
SSRI exposure	0.07	0.21	139.35	0.35	0.727	−0.34	0.49
Interaction (maternal mood at the third trimester × SSRI exposure)	−0.04	0.02	148.01	−1.86	0.066	−0.08	0.00

a. The estimates indicate differences in average internalising and anxiety behaviours over time between groups (SSRI exposure, yes/no) and differences in maternal mood at the third trimester; *n* = 160, those with data from to or more time points.

#### Hierarchical regressions

To gauge the relative contributions of maternal mood at the third trimester, current maternal mood and SSRI exposure status to internalised behaviours at each point over time (which is not possible using multilevel mixed model analyses), three hierarchical regressions were conducted with internalising behaviour scores at 3, 6 and 12 years as the dependent variable. Results are detailed in Table 5. At 3 years, the age of the children was entered in step 1 and was not statistically significant. For step 2, maternal mood at the third trimester was statistically significant and accounted for an additional 8.8% of unique variance in internalising behaviours. For step 3, maternal mood at 3 years was statistically significant and accounted for an additional 4.6% of unique variance. For step 4, the prenatal SSRI exposure's contribution to the model was not statistically significant.

**Table 5:** Hierarchical regression analyses predicting internalising behaviours across childhood

Step variables	β	Δ*R*^2^	Δ*F*	d.f.
**Model 1: Internalising behaviours at 3 years**				
Step 1		0.02	2.42	1, 145
Child age at 3 years	0.13			
Step 2		0.09	14.09***	1, 144
Child age at 3 years	0.06			
Maternal mood at the third trimester	0.30***			
Step 3		0.05	7.80**	1, 143
Child age at 3 years	0.02			
Maternal mood at the third trimester	0.18*			
Maternal mood at 3 years	0.25**			
Step 4		0.00	0.04	1, 142
Child age at 3 years	0.02			
Maternal mood at the third trimester	0.18			
Maternal mood at 3 years	0.25**			
SSRI exposure	0.02			
**Model 2: Internalising behaviours at 6 years**				
Step 1		0.04	5.86*	1, 139
Child age at 6 years	0.20*			
Step 2		0.13	20.80***	1, 138
Child age at 6 years	0.22**			
Maternal mood at the third trimester	0.36***			
Step 3		0.00	0.26	1, 137
Child age at 6 years	0.21*			
Maternal mood at the third trimester	0.33**			
Maternal mood at 6 years	0.05			
Step 4		0.00	0.05	1, 136
Child age at 6 years	0.20*			
Maternal mood at the third trimester	0.32**			
Maternal mood at 6 years	0.05			
SSRI exposure	0.02			
**Model 3: Internalising behaviours at 12 years**				
Step 1		0.00	0.15	1, 108
Child age at 12 years	−0.04			
Step 2		0.18	23.37***	1, 107
Child age at 12 years	0.02			
Maternal mood at the third trimester	0.43***			
Step 3		0.02	2.64	1, 106
Child age at 12 years	0.01			
Maternal mood at the third trimester	0.36***			
Maternal mood at 12 years	0.16			
Step 4		0.01	1.27	1, 105
Child age at 12 years	−0.01			
Maternal mood at the third trimester	0.30**			
Maternal mood at 12 years	0.15			
SSRI exposure	0.12			

SSRI, selective serotonin reuptake inhibitor.

* *P* < 0.05; **P < 0.01; ***P < 0.001.

At 6 years, child age was entered in step 1 and was statistically significant. For step 2, maternal mood at the third trimester was statistically significant and accounted for an additional 12.6% of unique variance in internalising behaviours. For step 3, maternal mood at 6 years was not statistically significant. For step 4, SSRI exposure was not statistically significant.

At 12 years, child age was entered in step 1 and was not statistically significant. For step 2, maternal mood at the third trimester continued to have a statistically significant impact and accounted for an additional 17.9% of unique variance in internalising behaviours. For step 3, maternal mood at 12 years was not statistically significant. For step 4, SSRI exposure was not statistically significant.

### Children's anxiety behaviours

Multilevel mixed model analyses (*n* = 160) investigated the impact of prenatal maternal mood, SSRI exposure and the interaction between prenatal maternal mood and SSRI exposure. Results showed that maternal mood during the third trimester was associated with increased child anxiety behaviour over time (Table 4). Neither SSRI exposure alone nor the interaction with prenatal maternal mood contributed significantly to the model.

#### Hierarchical regressions

To assess the relative contributions of maternal mood during the third trimester, current maternal mood and SSRI exposure status to anxiety behaviours at each point over time (which is not possible using multilevel mixed model analyses), three hierarchical regressions were conducted with anxiety behaviour scores at 3, 6 and 12 years as the dependent variable. Results are detailed in Table 6. At 3 years, the age of the children was entered in step 1 and was not statistically significant. For step 2, maternal mood at the third trimester was statistically significant and accounted for an additional 4.5% of unique variance. For step 3, maternal mood at 3 years was not statistically significant. For step 4, SSRI exposure was not statistically significant.

**Table 6:** Hierarchical regression analyses predicting anxiety behaviours across childhood

Step variables	β	Δ*R*^2^	Δ*F*	d.f.
**Model 1: Anxiety behaviours at 3 years**				
Step 1		0.00	0.33	1, 145
Child age at 3 years	−0.05			
Step 2		0.05	6.34**	1, 144
Child age at 3 years	−0.09			
Maternal mood at the third trimester	0.22**			
Step 3		0.01	1.48	1, 143
Child age at 3 years	−0.11			
Maternal mood at the third trimester	0.16			
Maternal mood at 3 years	0.12			
Step 4		0.00	0.29	1, 142
Child age at 3 years	−0.10			
Maternal mood at the third trimester	0.18			
Maternal mood at 3 years	0.12			
SSRI exposure	−0.05			
**Model 2: Anxiety behaviours at 6 years**				
Step 1		0.03	3.65	1, 139
Child age at 6 years	0.16			
Step 2		0.07	10.80**	1, 138
Child age at 6 years	0.17*			
Maternal mood at the third trimester	0.27**			
Step 3		0.01	1.16	1, 137
Child age at 6 years	0.15			
Maternal mood at the third trimester	0.21*			
Maternal mood at 6 years	0.11			
Step 4		0.00	0.41	1, 136
Child age at 6 years	0.14			
Maternal mood at the third trimester	0.19			
Maternal mood at 6 years	0.10			
SSRI exposure	0.06			
**Model 3: Anxiety behaviours at 12 years**				
Step 1		0.00	0.00	1, 108
Child age at 12 years	0.00			
Step 2		0.10	11.58**	1, 107
Child age at 12 years	0.05			
Maternal mood at the third trimester	0.32**			
Step 3		0.02	2.24	1, 106
Child age at 12 years	0.03			
Maternal mood at the third trimester	0.25^1^			
Maternal mood at 12 years	0.15			
Step 4		0.00	0.60	1, 105
Child age at 12 years	0.02			
Maternal mood at the third trimester	0.21			
Maternal mood at 12 years	0.15			
SSRI Exposure	0.08			

SSRI, selective serotonin reuptake inhibitor.

* *P* < 0.05; **P < 0.01; ***P < 0.001.

At 6 years, child age was entered in step 1 and was not statistically significant. For step 2, maternal mood during the third trimester was statistically significant and accounted for an additional 7.1% of unique variance. For step 3, maternal mood at 6 years was not statistically significant. For step 4, SSRI exposure was not statistically significant.

At 12 years, child age was entered in step 1 and was not statistically significant. For step 2, maternal mood at the third trimester was statistically significant and accounted for an additional 9.8% of unique variance. For step 3, maternal mood at 12 years was not statistically significant. For step 4, SSRI exposure was not statistically significant.

## Discussion

In this study we examined longitudinal patterns of child internalising and anxious behaviours from 3 to 12 years of age and associations with maternal depressive symptoms and prenatal SSRI treatment. As expected, prenatal maternal depressive symptoms, not prenatal SSRI exposure, were associated with persistently higher levels of internalising and anxiety behaviours from toddlerhood into pre-adolescence. Additional analyses examined the relative contribution of prenatal maternal depressive symptoms, current maternal depressive symptoms and prenatal SSRI treatment. We showed that prenatal maternal depressive symptoms accounted for more variability in child internalising and anxiety behaviours than current depressive symptoms at each child assessment or prenatal SSRI exposure.

Interestingly, a greater proportion of the children of mothers with prenatal SSRI treatment had anxiety symptoms in the clinical range (29.7%) at 12 years of age, compared with children without prenatal SSRI exposure (10.9%), according to the child self-report SCARED and maternal report on the CBCL. Even with SSRI treatment, pregnant women in our study remained symptomatic (mean HAMD score of 11) during the third trimester, raising the possibility of an apparent ‘pharmacotherapeutic failure’ which put children of SSRI-treated mothers at an additional developmental risk. However, further exploration of the nature of pharmacotherapeutic failure and a discussion on whether there was a negative impact of ‘unneeded SSRI exposure’ is beyond the scope of this paper.

Our findings are consistent with previous cohort-based studies investigating relationships between internalising behaviours and prenatal SSRI exposure^16–18^ and prenatal antidepressant exposure^15,20^ in 3- to 6-year-olds. Our outcomes related to anxiety behaviours are also consistent with a larger population-based study of prenatal SSRI exposure and later diagnosis of anxiety with children up to 14 years of age.^22^ In contrast, Lupattelli et al.^4^ found that SSRI exposure in late pregnancy (after week 29) was associated with increased levels of anxiety behaviours in 5-year-olds when prenatal maternal depressive and anxiety symptoms were controlled for. These contradictory findings may reflect methodological differences due to varying extents of prenatal exposure in late pregnancy (after week 29) and controlling for maternal anxiety symptoms in addition to depressive symptoms. As the majority of prenatal SSRI exposure studies have examined internalising behaviours, very little attention has been paid to anxiety behaviours as a specific behavioural phenotype. To date despite the determined efforts to control for the confounding effects of maternal mental health (pre- and postnatal and current) and other factors that potentially contribute to residual confounding (e.g. genetic inheritance, environment), precise associations and underlying neurodevelopmental mechanisms that explain associations between prenatal SSRI exposure and behavioural outcomes remain to be determined.

Together, findings from this longitudinal birth cohort study indicate that children with prenatal SSRI exposure are not at greater risk for having higher levels of internalising and anxious behaviours across childhood and into early adolescence. However, maternal prenatal depressed mood as well as current maternal mood across childhood persistently accounted for most of the variance in child behavioural outcomes. It is worth noting that mean maternal HAMD scores for the current study (≤11) were not in the clinical range for scores for moderate depression (17−23),^36^ which is consistent with other studies in mothers with prenatal SSRI treatment.^17,18^ With this in mind we need to ask whether any observed developmental associations with prenatal SSRI exposure reflects a sustained effect related to *in utero* exposure to SSRIs or whether SSRI exposure merely represents a ‘proxy exposure’ that accompanies maternal mood disturbances reflecting the possible influence of genetic and environmental factors that may also contribute to child development. Importantly, our findings are consistent with a growing literature that increasingly shows that, regardless of SSRI exposure, maternal mood continues to influence behaviour across childhood,^5^ highlighting that regardless of maternal prenatal SSRI treatment, development in children of depressed mothers treated with an SSRI during pregnancy remains disproportionality at risk. These findings have critical implications for ensuring timely and effective interventions for mothers during and following pregnancy as well as long-term follow-up for their children. In this study we observed that all measures of maternal depressive symptoms were associated with both internalising and anxiety behaviours at 12 years and point to the importance of ongoing assessments of maternal mental health long after delivery. Additional longitudinal studies are needed to determine risks related to perinatal maternal depressive symptoms and identify who can benefit from maternal pre- and postnatal antidepressant treatment.

### Limitations

A few key limitations for this study are worth noting. First, the majority of mothers in our cohort were highly educated, and therefore these effects may not generalise across the full socioeconomic spectrum. Maternal education is only one component of socioeconomic status (SES) and other indicators (e.g. occupation, income, marital status) are needed to establish a broader perspective on the influence of SES. Second, as the measures of internalising and anxiety behaviours were completed by mothers, mothers who were depressed at the time may have been more likely to have a bias in over-reporting these behaviours. However, we examined this possibility using additional analyses comparing maternal reports of their child's anxiety and the children's self-reports using the SCARED and they were significantly associated, suggesting that maternal reports of anxiety were accurate. Third, as is typical in longitudinal research, some participants declined to participate over time, so our original sample of 191 participants had reduced to 118 at 12 years (62%). We therefore acknowledge an inherent selection bias (uncontrolled or even unmeasurable) that emerged over time and the small sample. Although we did conduct scenario-based quantitative bias analysis to partly address this concern, future research is still needed with larger samples to confirm these results.

Finally, although this was a longitudinal prospective study starting in the second trimester, yet unmeasured factors related to maternal illness severity, genetics and the everyday environment that could also have contributed to the impact of exposure to maternal mood and SSRI antidepressants need to be considered by future researchers. For example, some studies have found that genetic variations were associated with poor, rapid or ultrarapid metabolism, which could have an impact on the effectiveness of an SSRI dose.^39^ Owing to our sample size, we had to limit the number of covariates used in the study; however, it is possible that studies in larger populations with sufficient statistical power might be able to address this limitation in future research.

In conclusion, this prospective longitudinal study found prenatal maternal depressive symptoms, not prenatal SSRI exposure per se, was associated with persistently increased levels of internalizing and anxiety behaviours from 3 to 12 years of age. These findings highlight that regardless of prenatal SSRI treatment, behavioural risk for affective disturbances associated with perinatal mood disturbances continued into early adolescence.

## Data Availability

The data that support the findings of this study are available on request from T.F.O. (toberlander@cw.bc.ca). The data are not publicly available owing to privacy/ethical restrictions.

## Supplementary material

For supplementary material accompanying this paper visit http://doi.org/10.1192/bjo.2022.623. click here to view supplementary material

## References

[1] Pawluski JL, Lonstein JS, Fleming AS. The neurobiology of postpartum anxiety and depression. Trends Neurosci 2017; 40: 106–20. 28129895 10.1016/j.tins.2016.11.009

[2] Brandlistuen RE, Ystrom E, Eberhard-Gran M, Nulman I, Koren G, Nordeng H. Behavioural effects of fetal antidepressant exposure in a Norwegian cohort of discordant siblings. Int J Epidemiol 2015; 44: 1397–407. 25873178 10.1093/ije/dyv030 PMC4588862

[3] Hanley GE, Brain U, Oberlander TF. Prenatal exposure to serotonin reuptake inhibitor antidepressants and childhood behavior. Pediatr Res 2015; 78: 174–80. 25897539 10.1038/pr.2015.77

[4] Lupattelli A, Wood M, Ystrom E, Skurtveit S, Handal M, Nordeng H. Effect of time-dependent selective serotonin reuptake inhibitor antidepressants during pregnancy on behavioral, emotional, and social development in preschool-aged children. J Am Acad Child Adolesc Psychiatry 2018; 57: 200–8. 29496129 10.1016/j.jaac.2017.12.010 PMC5843872

[5] Rommel AS, Bergink V, Liu X, Munk-Olsen T, Molenaar NM. Long-term effects of intrauterine exposure to antidepressants on physical, neurodevelopmental, and psychiatric outcomes: a systematic review. J Clin Psychiatry 2020; 81(3): 19r12965. 10.4088/JCP.19r12965 PMC8739257 32412703

[6] Ewing G, Tatarchuk Y, Appleby D, Schwartz N, Kim D. Placental transfer of antidepressant medications: implications for postnatal adaptation syndrome. Clin Pharmacokinet 2015; 54: 359–70. 25711391 10.1007/s40262-014-0233-3 PMC4566926

[7] Kim J, Riggs KW, Misri S, Kent N, Oberlander TF, Grunau RE, Stereoselective disposition of fluoxetine and norfluoxetine during pregnancy and breast-feeding. Br J Clin Pharmacol 2006; 61: 155–63. 16433870 10.1111/j.1365-2125.2005.02538.x PMC1885002

[8] Kalueff AV, Olivier JD, Nonkes LJ, Homberg JR. Conserved role for the serotonin transporter gene in rat and mouse neurobehavioral endophenotypes. Neurosci Biobehav Rev 2010; 34: 373–86. 19698744 10.1016/j.neubiorev.2009.08.003

[9] Ansorge MS, Zhou M, Lira A, Hen R, Gingrich JA. Early-life blockade of the 5-HT transporter alters emotional behavior in adult mice. Science 2004; 306: 879–81. 15514160 10.1126/science.1101678

[10] Homberg JR, Schubert D, Gaspar P. New perspectives on the neurodevelopmental effects of SSRIs. Trends Pharmacol Sci 2010; 31: 60–5. 19963284 10.1016/j.tips.2009.11.003

[11] Hutchison SM, Mâsse LC, Pawluski JL, Oberlander TF. Perinatal selective serotonin reuptake inhibitor (SSRI) and other antidepressant exposure effects on anxiety and depressive behaviors in offspring: a review of findings in humans and rodent models. Reprod Toxicol 2021; 99: 80–95. 33253794 10.1016/j.reprotox.2020.11.013

[12] Hermansen TK, Røysamb E, Augusti EM, Melinder A. Behavior and inhibitory control in children with prenatal exposure to antidepressants and medically untreated depression. Psychopharmacology 2016; 233: 1523–35. 26924747 10.1007/s00213-016-4248-3

[13] Rommel AS, Momen NC, Molenaar NM, Liu X, Munk-Olsen T, Bergink V. Long-term prenatal effects of antidepressant use on the risk of affective disorders in the offspring: a register-based cohort study. Neuropsychopharmacology 2021; 46(8): 1518–25. 33820955 10.1038/s41386-021-01005-6 PMC8209173

[14] Oberlander TF, Papsdorf M, Brain UM, Misri S, Ross C, Grunau RE. Prenatal effects of selective serotonin reuptake inhibitor antidepressants, serotonin transporter promoter genotype (SLC6A4), and maternal mood on child behavior at 3 years of age. Arch Pediatr Adolesc Med 2010; 164: 444–51. 20439795 10.1001/archpediatrics.2010.51

[15] Grzeskowiak LE, Morrison JL, Henriksen TB, Bech BH, Obel C, Olsen J, Prenatal antidepressant exposure and child behavioural outcomes at 7 years of age: a study within the Danish National Birth Cohort. BJOG 2016; 123: 1919–28. 26374344 10.1111/1471-0528.13611

[16] Misri S, Reebye P, Kendrick K, Carter D, Ryan D, Grunau RE, Internalizing behaviors in 4-year-old children exposed in utero to psychotropic medications. Am J Psychiatry 2006; 163: 1026–32. 16741203 10.1176/ajp.2006.163.6.1026

[17] Nulman I, Koren G, Rovet J, Barrera M, Pulver A, Streiner D, Neurodevelopment of children following prenatal exposure to venlafaxine, selective serotonin reuptake inhibitors, or untreated maternal depression. Am J Psychiatry 2012; 169: 1165–74. 23128923 10.1176/appi.ajp.2012.11111721

[18] Nulman I, Koren G, Rovet J, Barrera M, Streiner DL, Feldman BM. Neurodevelopment of children prenatally exposed to selective reuptake inhibitor antidepressants: Toronto sibling study. J Clin Psychiatry 2015; 76: e842–7. 26231010 10.4088/JCP.14m09240

[19] Marroun H E, White T, Verhulst FC, Tiemeier H. Maternal use of antidepressant or anxiolytic medication during pregnancy and childhood neurodevelopmental outcomes: a systematic review. Eur Child Adolesc Psychiatry 2014; 23: 973–92. 24863148 10.1007/s00787-014-0558-3

[20] Pedersen LH, Henriksen TB, Bech BH, Licht RW, Kjaer D, Olsen J. Prenatal antidepressant exposure and behavioral problems in early childhood – a cohort study. Acta Psychiatr Scand 2013; 127: 126–35. 23126521 10.1111/acps.12032

[21] Lupattelli A, Wood M, Ystrom E, Skurtveit S, Handal M, Nordeng H. Effect of time-dependent selective serotonin reuptake inhibitor antidepressants during pregnancy on behavioral, emotional, and social development in preschool-aged children. J Am Acad Child Adolesc Psychiatry 2018; 57: 200–8. 29496129 10.1016/j.jaac.2017.12.010 PMC5843872

[22] Malm H, Brown AS, Gissler M, Gyllenberg D, Hinkka-Yli-Salomäki S, McKeague IW, Gestational exposure to selective serotonin reuptake inhibitors and offspring psychiatric disorders: a national register-based study. J Am Acad Child Adolesc Psychiatry 2016; 55: 359–66. 27126849 10.1016/j.jaac.2016.02.013 PMC4851729

[23] Campbell KS, Collier AC, Irvine MA, Brain U, Rurak DW, Oberlander TF, Maternal serotonin reuptake inhibitor antidepressants have acute effects on fetal heart rate variability in late gestation. Front Psychiatry 2021; 12: 680177. 10.3389/fpsyt.2021.680177 PMC8415315 34483982

[24] Lash TL, Fox MP, Fink AK. Applying Quantitative Bias Analysis to Epidemiologic Data. Springer, 2009.

[25] Kessler RC, Chiu WT, Demler O, Walters EE. Prevalence, severity, and comorbidity of 12-month DSM-IV disorders in the National Comorbidity Survey Replication. Arch Gen Psychiatry 2005; 62: 617–27. 15939839 10.1001/archpsyc.62.6.617 PMC2847357

[26] Hamilton M. A rating scale for depression. J Neurol Neurosurg Psychiatry 1960; 23: 56–62. 14399272 10.1136/jnnp.23.1.56 PMC495331

[27] Beck AT, Epstein N, Brown G, Steer RA. An inventory for measuring clinical anxiety: psychometric properties. J Consult Clin Psychol 1988; 56: 893–7. 3204199 10.1037//0022-006x.56.6.893

[28] Brown C, Schulberg HC, Madonia MJ. Assessment [of] depression in primary care practice with the Beck Depression Inventory and the Hamilton Rating Scale for Depression. Psychol Assess 1995; 7: 59–65.

[29] Achenbach T. Manual for the Child Behavior Checklist 2/3 Years. University of Vermont, Department of Psychiatry, 1992.

[30] Achenbach T, Rescorla L. Manual for the ASEBA School-Age Forms & Profiles: An Integrated System of Multi-Informant Assessment. University of Vermont. Research Center for Children, Youth, & Families, 2001, 1617.

[31] Berubé R. Bibliography of Published Studies Using the Achenbach System of Empirically Based Assessment: 2006 Edition. Research Center for Children, Youth, & Families, University of Vermont, 2010.

[32] Boyle MH, Offord DR, Racine Y, Fleming JE, Szatmari P, Sanford M. Evaluation of the revised Ontario Child Health Study scales. J Child Psychol Psychiatry 1993; 34: 189–213. 8444992 10.1111/j.1469-7610.1993.tb00979.x

[33] Lemery-Chalfant K, Schreiber JE, Schmidt NL, van Hulle CA, Essex MJ, Goldsmith HH. Assessing internalizing, externalizing, and attention problems in young children: validation of the MacArthur HBQ. J Am Acad Child Adolesc Psychiatry 2007; 46: 1315–23. 17885573 10.1097/chi.0b013e3180f616c6

[34] Birmaher B, Brent DA, Chiappetta L, Bridge J, Monga S, Baugher M. Psychometric properties of the Screen for Child Anxiety Related Emotional Disorders (SCARED): a replication study. J Am Acad Child Adolesc Psychiatry 1999; 38: 1230–6. 10517055 10.1097/00004583-199910000-00011

[35] Gueorguieva R, Krystal JH. Move over ANOVA: progress in analyzing repeated-measures data and its reflection in papers published in the *Archives of General Psychiatry*. Arch Gen Psychiatry 2004; 61: 310–7. 14993119 10.1001/archpsyc.61.3.310

[36] Zimmerman M, Martinez JH, Young D, Chelminski I, Dalrymple K. Severity classification on the Hamilton depression rating scale. Journal of Affective Disorders 2013; 150: 384–8. 23759278 10.1016/j.jad.2013.04.028

[37] McGraw KO, Wong SP. Forming inferences about some intraclass correlation coefficients. Psychol Methods 1996; 1: 30–46.

[38] Bartko JJ. The intraclass correlation coefficient as a measure of reliability. Psychol Rep 1966; 19: 3–11. 5942109 10.2466/pr0.1966.19.1.3

[39] Altar CA, Hornberger J, Shewade A, Cruz V, Garrison J, Mrazek D. Clinical validity of cytochrome P450 metabolism and serotonin gene variants in psychiatric pharmacotherapy. Int Rev Psychiatry 2013; 25: 509–33. 24151799 10.3109/09540261.2013.825579

[40] Cohen J. Statistical Power Analysis (2nd edn). Erlbaum, 1988.

